# Radiation Pneumonitis after Intensity-Modulated Radiotherapy for Esophageal Cancer: Institutional Data and a Systematic Review

**DOI:** 10.1038/s41598-018-38414-5

**Published:** 2019-02-19

**Authors:** J. J. Tonison, S. G. Fischer, M. Viehrig, S. Welz, S. Boeke, K. Zwirner, B. Klumpp, L. H. Braun, D. Zips, C. Gani

**Affiliations:** 1 Department of Radiation Oncology, University Hospital and Medical Faculty Tübingen, Eberhard Karls University Tübingen, Tübingen, Germany; 2 Department of Diagnostic and Interventional Radiology, Eberhard Karls University Tübingen, Tübingen, Germany; 3 Gastrointestinal Cancer Center, Comprehensive Cancer Center Tübingen-Stuttgart, Tübingen, Germany; 4 German Cancer Consortium (DKTK), Partner site Tübingen, Tübingen, Germany; 5 German Cancer Research Center (DKFZ), Heidelberg, Germany

## Abstract

Radiation pneumonitis (RP) is a serious complication that can occur after thoracic radiotherapy. The goal of this study is to investigate the incidence of RP after radiochemotherapy with intensity modulated radiotherapy (IMRT) in patients with esophageal cancer and correlate this with dose volume histogram (DVH) related parameters. For this purpose, the clinical course of 73 patients was evaluated and irradiation doses to the lungs were extracted from radiotherapy treatment plans. Furthermore, a systematic review on this topic was conducted across PubMed. In our institutional cohort, Common Terminology Criteria for Adverse Events (CTCAE) grade II or higher RP occurred in four patients (5.5%). The systematic review identified 493 titles of which 19 studies reporting 874 patients qualified for the final analysis. No grade IV or V RP after radiochemotherapy with IMRT for esophageal cancer was reported in the screened literature. Grade II or higher RP is reported in 6.6% of the patients. A higher incidence can be seen with increasing values for lung V20. In conclusion, our institutional data and the literature consistently show a low incidence of symptomatic RP after radiochemotherapy in patients with esophageal cancer treated with IMRT. However, efforts should be made to keep the lung V20 below 23% and specific caution is warranted in patients with pre-existing lung conditions.

## Introduction

Due to the anatomical proximity to vital organs such as the lung and heart and the ability to achieve a high dose conformity in the tumor region, intensity modulated radiotherapy (IMRT) is increasingly used in patients with esophageal cancer^1,2^. One of the dose-limiting toxicities of thoracic radiotherapy is radiation pneumonitis (RP) characterized by dyspnea, dry cough and low-grade fever. RP can be categorized into two phases: the acute phase, which normally develops approximately one to three months post radiotherapy, and a chronic phase, which comprises of a deterioration of lung function and the onset of lung fibrosis^3^. While low grade and asymptomatic RP is a frequent finding on imaging studies and does not require intervention, acute symptomatic RP is commonly treated with corticosteroids and antimicrobials^4^.

Data suggests that both patient and treatment related factors such as fractionation, radiation dose, volume of treated lung and pre-existing lung diseases may increase the risk of RP after radiotherapy^5,6^. The vast majority of published data on radiation pneumonitis is derived from patients with lung malignancies treated with 3D conformal radiotherapy; the same applies to models used to predict RP^7,8^. However, disease and treatment specific, as well as anatomical, differences between lung and esophageal tumors limit the transferability of RP prediction models from pulmonary to esophageal tumors. Consensus guidelines providing recommendations for acceptable lung doses in the context of radiotherapy for esophageal cancer have not been established yet. Only limited data is available examining the incidence and dosimetric risk factors for RP in patients with esophageal cancer treated with IMRT. Therefore, the goal of the present study is to assess the incidence of RP in patients with esophageal cancer after radiochemotherapy with IMRT in an institutional cohort and to provide clinical recommendations based on this cohort and the aggregated data from a systematic review of the published literature.

## Methods

### Patients

A total of 85 patients with histologically confirmed esophageal cancer who received definitive or neoadjuvant radiochemotherapy at the University Hospital Tuebingen from 2006 to 2017 were included. Patients with cervical esophageal tumors were excluded if the planning target volume did not extend below the upper edge of the aortic arch in order to avoid inclusion of patients with an irrelevant pulmonary dose burden. A total of 73 patients had a follow-up with computed tomography (CT) imaging studies of at least three months. A summary of these 73 patients and treatment related parameters is shown in Table 1. Signs of pulmonary emphysema on CT imaging or pulmonary function tests were seen in 21% of the patients. The study was approved by the local Ethics Committee (Study ID: 223/2015R).

**Table 1:** Patient and treatment characteristics.

**Age mean(range)**	65	(44–79)
**Gender**	**n**	**%**
male	52	71
female	21	29
**cT category**		
1	1	1
2	11	15
3	53	73
4	8	11
**Primary tumor site**		
cervical	11	15
upper thoracic	21	29
mid thoracic	25	34
lower thoracic/GEJ	16	22
**Histology**		
squamous cell carcnoma	61	84
adenocarcinoma	11	15
other	1	1
**cN category**		
cN0	20	27
cN+	53	73
**RT concept**		
definitive	59	81
neoadjuvant	14	19
**IMRT technique**		
step and shoot	20	27
sliding window	9	12
VMAT	44	60

RT-radiotherapy, IMRT-Intensity modulated radiotherapy, VMAT-volumetric modulated arc therapy. The primary tumor site is defined by the center of the primary tumor. Due to rounding errors, single values might not sum up to 100%. GEJ – gastro-esophageal junction.

### Radiochemotherapy

Target volume definition was consistent with recently published consensus guidelines^9^. Deviations from this standard were made at the discretion of the treating radiation oncologist, e.g. in case of limited pulmonary function or cardiac comorbidities. Radiotherapy was applied either in a “step and shoot”, “sliding window” or “volume modulated arc therapy“ (VMAT) technique.

During the confined period, all 73 patients received concomitant radiochemotherapy. The majority of patients (59 patients, 80.8%) received definitive radiochemotherapy with a median dose of 60 Gy (range 50–70 Gy). A total of 14 patients (19.2%) underwent preoperative radiochemotherapy (median dose 45 Gy, range 41.4–50 Gy). Platinum and 5-fluorouracil based chemotherapy was given in most cases. Alternative chemotherapy regimens included mitomycin-C, docetaxel or cetuximab. Based on our institutional standard operating procedure, the following dose constraints were used: mean lung dose (MLD) < 20 Gy, relative volume of the lungs receiving 20 Gy (lung V20) < 30% and lung V30 < 18%, respectively.

For treatment planning, an in-house treatment planning system “Hyperion” (a progenitor of Monaco©, Elekta, Crawley, United Kingdom) was used.

### Grading of radiation pneumonitis

Symptoms of radiation pneumonitis were recorded according to the common toxicity criteria of adverse events (CTCAE), version 4.03. According to this classification, grade II pneumonitis is defined by the need of medical treatment or limitations in activities of daily life. All patient documents, including digital notes, consultations, radiological images, scanned medical records and reports were used to score toxicity. Scoring of RP was performed at the consensus of a radiologist (BK), pulmonologist (MV) and radiation oncologist (CG). Furthermore, the severity of RP on CT images was graded on a Likert scale from “0” (representing no signs of RP) to “4” (evidence of severe RP).

### Follow up

Follow-up visits included a medical history and physical examination, also addressing signs of radiation pneumonitis. Cross-sectional imaging was routinely performed in three monthly intervals at least during the first year of follow-up. The duration of follow-up ranged from four months to 92 months, mean and median follow-up was 25 months and 17 months, respectively. Fifty-six patients had a follow-up of at least 12 months.

### Literature review - Search strategy and selection criteria

We searched Pubmed using the search term “(IMRT OR VMAT OR Intensity OR Volumetric) AND (“esophageal cancer” OR “esophageal squamous*” OR “esophageal adeno*” OR “cancer of the esophagus”)” to retrieve studies regarding the incidence of RP after IMRT in esophageal cancer. Two searches were performed: The first search was on February 22^nd^ 2016 and an update on February 2^nd^ 2017. For inclusion in this systematic review, the studies had to be written in German or English language. Both prospective and retrospective studies were included. It was required that the incidence of RP after IMRT is reported; studies with mixed cohorts of 3D conventional radiotherapy and IMRT were included when the incidence of RP in the IMRT was reported separately. RP had to be specifically mentioned under toxicities; it was not concluded that no RP occurred when RP was not mentioned among toxicities.

### Statistical analysis

Dose-volume histogram (DVH) parameters were extracted from the corresponding DVH files of Hyperion by a Microsoft Excel template (Microsoft Corp., Redmond, Washington, USA). DVH parameters were tested for normal distribution with Kolmogorov-Smirnow Test and subsequently DVH parameters were compared between groups using Mann-Whitney’s U-Test in SPSS (IBM, Armonk, New York, USA).

## Results

### Dosimetric parameters

The MLD in all evaluated patients (n = 73) was 11.1 Gy, range (2.9 Gy – 17.7 Gy). Mean V5 (range) was 56.4% (14.3–99.4%), mean (range) for V10 38.9% (8.5–86.2%), mean (range) for V20 18.74% (3.7–31.0%) and mean (range) for V30 9.5% (1.37–19.6%).

As mentioned, twelve patients had a follow-up with imaging of less than three months and were therefore considered not evaluable for RP. Seven of these patients were lost to follow-up, two died of pneumonia after development of tracheobronchial fistulas, one patient of progressive disease, one of a tumor bleeding during treatment and one after stroke. Compared with evaluable patients who had a median follow-up with cross-sectional imaging of 15 months, no significant differences were seen regarding MLD, V5, V10, V20, V30 (Data not shown).

All patients were treated with concomitant radiochemotherapy. During follow-up, four patients (5.5%) out of 73 evaluated patients developed clinical signs of grade II acute RP. The first patient had received definitive radiochemotherapy with 50 Gy followed by a 10 Gy boost with brachytherapy. The patient had pre-existing but asymptomatic fibrotic pulmonary changes on CT, which had aggravated two months after radiochemotherapy and resulted in dyspnea, limiting instrumental activities of daily life. The patient received treatment with prednisone with subsequent improvement of symptoms. The remaining three patients had no pre-existing lung conditions. In these patients symptoms such as dyspnea or cough and a decrease in pulmonary function tests (forced expiratory volume in one second (FEV1) or diffusion capacity) prompted steroid treatment with subsequent symptom improvement.

Patients with grade II or higher RP did not have higher lung DVH parameters compared with patients who developed no more than grade I RP (V5 51.8% vs. 57.1%, V10 34.7% vs 39.4%, V20 18.6% vs. 18.8%, MLD 11.0% vs. 11.2%, p > 0.05 in all cases).

Another seven patients had pronounced changes (grade 3 or 4 on Likert scale) on CT-scans typical for RP, yet none of these patients showed any clinical signs of RP during follow up (CTCAE grade I). Of these seven patients, five had a follow-up of at least 12 months. The remaining two patients were both diagnosed with distant metastases four months after the end of radiotherapy and died of progressive disease within a few weeks. The highest V5 among the patients developing CTCAE grade II was 70.6%, none of the patients with a higher V5 (n = 12) developed CTCAE grade II or higher RP. No patient with pulmonary emphysema developed symptomatic RP.

### Literature review

**Figure 1:**
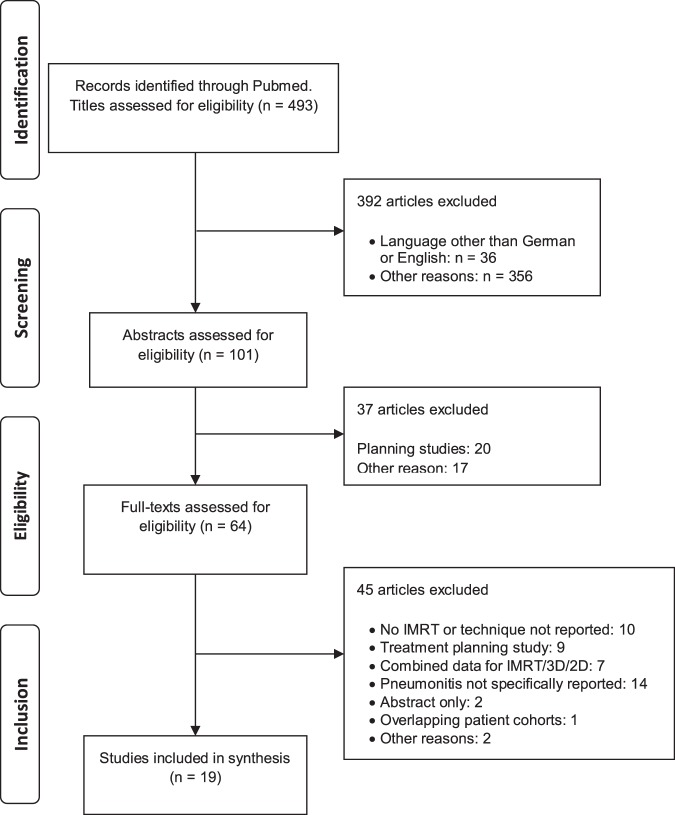
“Preferred Reporting Items for Systematic Reviews and Meta-Analyses” (PRISMA) flowchart depicting the selection process of studies that reported on the incidence of radiation pneumonitis after intensity modulated radiotherapy (IMRT).

**Figure 2:**
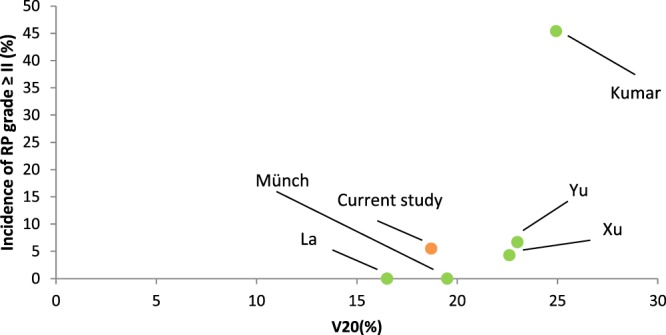
Incidence of grade II or higher radiation pneumonitis (RP) in dependence of the applied V20 to the lungs.

A PRISMA (Preferred Reporting Items for Systematic Reviews and Meta-Analyses) flowchart is shown in Fig. 1^10^. In the first step 493 titles were screened and studies obviously not meeting inclusion criteria were excluded. A total of 101 abstracts were reviewed, leaving 64 full text articles, each reviewed for eligibility by two or more co-authors (JT, CG, KZ, SB, SW, LHB). In selected cases, corresponding authors were contacted, for instance when overlapping patient cohorts were suspected. Finally, 19 full articles with a total of 874 patients were included in the final dataset (Table 2). The largest study with 228 patients included patients who received radiotherapy only postoperatively. Eleven of the 19 studies with a total of 590 (68%) patients were conducted in Asia. Fourteen studies were retrospective. Grading of RP was performed according to the Common Terminology Criteria of Adverse Events (CTCAE) in its third or forth version and according to the “Radiation Therapy Oncology Group” RTOG criteria. While slight differences exist between grading systems, all are five-tiered. Grade I generally implies imaging findings without symptoms or only mild symptoms that do not require medical intervention^11–13^. No study reported grade IV or grade V RP, however three studies grouped different grades of RP as “≥ grade II” or reported only an overall rate of RP^14–16^. Grade I or II RP was the highest reported grade of RP in 13 of 19 studies. In trials that specifically reported grade II or higher RP, 54 of 818 (6.6%) patients had at least grade II RP. Kumar *et al*. observed the highest incidence of grade II pneumonitis with 41% and 4.5% grade III RP, graded according to CTCAE, version 3^17^. The V20 in the Kumar trial was the highest reported (V20 = 24.93%) but other studies with only slightly lower values for V20 had lower rates of RP. Using the same grading system in a prospective trial, Yu *et al*. report no grade III or grade IV RP and 6.7% grade II RP. The median V20 in that trial was 23.6%^18^. Hsieh *et al*. observed no grade III or IV RP according to the RTOG grading system in 39 patients treated with definitive radiochemotherapy and an applied mean V20 of 23.4%^19^. A correlation of V20 with the incidence of RP is shown in Fig. 2. A wide range was seen for the lung V5. The mean V5 in the trial by Münch *et al*. was as high as 90.1%, however no “treatment of symptomatic pneumonitis” was reported^20^.

**Table 2:** Summary of all studies that qualified for the qualitative and quantitative analysis after systematic review.

First author	Year	Region	Study design	Total patient n	Patients with IMRT n	Inclusion limited to specific esophageal sites?	Radiotherapy indication	Conc. Chx	Prescribed lung dose constraints	Irradiated lung doses	Time of first follow-up	Grading of lung toxicity according to	Incidence of pneumonitis (only for IMRT patients)
Castillo^14^	2015	USA	retr	228	135	no	nr	yes	nr	nr for IMRT	within 6 months	CTCAE 4.03	≥ grade II: 14.8%
Fu^29^	2014	China	pros	25	25	no	def	yes	V20 ≤ 30%	nr	2 months	RTOG	grade I: 8%, grade II: 4%
Gerber^42^	2014	USA	retr	41	41	GEJ and thoracic	def, pre-OP	both	V20 ≤ 20%, V30 ≤ 15%, V40 ≤ 10%	nr	3 months	CTC3	grade II: 2,4%, grade III: 2,4%
Hsieh^19^	2016	Taiwan	retr	39	39	no	def	yes	MLD < 15 Gy, V20 < 30%	V5 = 67.8%, V20 = 23.41%	2–12 weeks	RTOG	grade III/IV: 0%
Kumar^17^	2011	India	pros	45	22	no	def, pre-OP, post-OP	yes	nr	V5 = 59.80%, V10 = 46.43%, V20 = 24.93%, V30 = 8.57%	1 month	CTCAE 3	grade II 9/22, grade III 1/22
La^43^	2010	USA	retr	30	30	upper, mid, lower, GEJ	def, pre-OP	yes	V20 < 20%, V30 < 15%, MLD < 15 Gy	V5 = 53%, V20 = 16.5%, V30 = 5.9%, MLD = 10.9 Gy (all median)	6 weeks	CTCAE 3	0% (grade not specified)
Li^44^	2016	China	pros	15	15	no	def	yes	V20 ≤ 30%	nr	6–8 weeks	CTCAE 3	grade I: 6,7%
Li^45^	2011	China	pros	12	12	Thoracic	def	yes	V20 ≤ 30%	nr	6–8 weeks	CTCAE 3	grade I: 16,7%
Münch^20^	2016	Germany	retr	37	17	mid and lower thoracic	pre-OP	yes	nr	V5 = 90.1%, V10 = 68.2%, V20 = 19.5%	within 3 months	CTC 4.03	“no treatment of pneumonitis recorded”
Nguyen^46^	2014	USA	retr	10	10	no	def, post-OP	yes	V5 < 50%, V10 < 40%, V15 < 30%, V20 < 25%	V5 = 50%, V10 = 38%, V15 = 25%, V20 = 18%*	1 month	RTOG	grade III/IV: 0%
Roeder^47^	2014	Germany	retr	27	27	no	def	yes	nr	nr	nr	CTCAE V3.0	“symptomatic pneumonitis 3.7%“
Tu^48^	2013	China	retr	36	36	cervical and upper thoracic	def	yes	V20 ≤ 30%, MLD ≤ 15 Gy	nr	1 month	CTCAE 3	0% (grade not specified)
Wang^49^	2006	USA	retr	7	7	cervical and upper thoracic	def	yes	V20 < 40%	V20 = 10.3–36%, MLD = 12.6 Gy (median)	1 month	RTOG	0% symptomatic RP, 29% radiographic changes
Xu^50^	2016	China	retr	69	69	no	def	both	V20 ≤ 34%, MLD < 17 Gy	V20 = 22.6%, MLD 13.4 Gy	1 month	CTC 4.0	grade I: 15.9%, grade II: 4.3%
Yu^18^	2014	China	pros	45	45	no	def	both	V20 ≤ 25%, MLD ≤ 15 Gy	V20 = 23% (median), MLD 13 Gy (median)	1 month	CTCAE 3	grade II: 6.7%
Zeng^16^	2016	USA	retr	17	17	no	pre-OP	yes	nr	nr	nr	nr	any grade: 11.7%
Zhang^15^	2015	China	retr	228	228	thoracic	post-OP	both	V20 < 28%	nr	3–6 months	nr	≥ grade II: 5.7%
Zhao^28^	2016	China	pros	21	21	cervical, upper, mid, lower thoracic	def	yes	nr	nr	nr	nr	grade I: 4.8%, grade II 4.8%
Zhu^51^	2013	China	retr	78	78	cervical, upper, mid, lower thoracic	def	yes	nr	V20 ≤ 25%	3 months	CTCAE 3	grade I: 2.6%

IMRT – Intensity modulated radiotherapy, conc. Chx – concomitant chemotherapy, retr – retrospective, pros – prospective, GEJ – gastro-esophageal junction, def – definitive, pre-OP – pre-operative, post-OP – post-operative, nr – not reported, Vx – Volume of bilateral lungs receiving x Gray, MLD – mean lung dose, Gy – Gray, RTOG – radiation therapy oncology group, CTC – common toxicity criteria, CTCAE – common terminology criteria for adverse events. *Data reported for a single patient.

## Discussion

The aim of this study was to evaluate the incidence of RP in patients with esophageal cancer treated with IMRT and concomitant chemotherapy. For this purpose, we screened our institutional cohort and performed a systematic review of the available literature summarizing the outcome for a total of 874 patients. We furthermore correlated DVH parameters with the development of RP. In our cohort only four of 73 patients developed RP requiring medical intervention with steroid treatment and antibiotics leading to recovery in all cases. This low incidence and the absence of any high grade RP is in line with the data from our systematic review.

Neither our institutional data nor data from the systematic review suggest any relevance of the low dose lung DVH parameters, in particular of lung V5. Compared with conventional 3D planned radiotherapy, IMRT can increase target conformality and decrease high dose volumes in organs at risk at the expense of a higher low dose burden^21–23^. After the introduction of IMRT into clinical practice, concerns about these low dose areas emerged as some studies had suggested a potential negative impact of these low-dose areas in terms of RP^24–26^. However, the majority of trials were not able to reproduce this observation. On the contrary, a recent secondary analysis of the RTOG 0617 trial showed a significantly lower incidence of grade III or higher RP in patients who were treated with IMRT for non-small cell lung cancer compared with 3D conventional radiotherapy (3.5% vs. 7.9%) despite a significantly higher V5 in the IMRT group (61.6% vs. 54.8%). No difference regarding survival endpoints was seen^27^. In the case of esophageal cancer, confirmatory results are seen in our institutional data in which no patient with a V5 greater than 71% developed grade II or higher RP. A similar observation was made in a cohort by Münch and colleagues who observed no clinical signs of RP during follow-up despite a mean V5 of 90.1%, which is the highest mean V5 reported in our review^20^. Regarding V20, which was the most frequently reported DVH parameter, Fig. 2 suggests a strong dose effect with a steep increase in the incidence of symptomatic RP between 23%-25%. Since only one trial reported a V20 greater than 23%, a sigmoid curve that would demonstrate the steepness of this effect could not be generated. Based on latter observations, we recommend limiting the V20 to 23% or lower in order to keep the risk for grade II or higher RP below 10%. Efforts to lower the V20 should clearly be prioritized over lowering the V5.

Our review also included phase I/II studies investigating novel radiotherapy-chemotherapy combinations. For example, Fu *et al*. studied the combination of radiotherapy with cisplatin and pemetrexed in esophageal squamous cell carcinoma. In this study and similarly in Zhao *et al*. who tested the incorporation of erlotinib into definitive radiochemotherapy, no excessive incidence of RP was seen^28,29^.

Many studies have looked into different risk factors predisposing patients to develop RP after radiotherapy. While there is conflicting data regarding pulmonary emphysema as a risk factor for RP, the literature is more consistent in terms of pre-existing fibrotic changes, which have been shown to be a critical predictor of severe RP^30–33^. In our cohort, no patient with pulmonary emphysema developed symptomatic RP, but the most severe case of RP was seen in a patient with pre-exiting fibrosis. Furthermore, Hope *et al*. showed that inferior tumor position was strongly correlated with RP in their series of 219 patients^34^. Other studies suggest that additional factors need to be considered to determine the risk of radiation pneumonitis, including genetic and immunologic parameters^26,35–38^.

The primary tumor remains a frequent site of failure after definitive radiochemotherapy in esophageal cancer. In order to tackle this issue, the randomized Intergroup 0123 trial had tested dose escalation to 64.8 Gy compared with 50.4 Gy. No benefit of dose-escalation was seen and, surprisingly, the higher rate of treatment associated deaths reported in the 64.8 Gy arm was mostly due to events that occurred prior to the boost^39^. It should be noted, that advanced radiotherapy techniques were not used in this trial. Planning studies have shown that the use of IMRT with a simultaneous integrated boost can facilitate a dose escalation up to 64.8 Gy without increasing doses to organs at risk such as the heart and lung compared with 2D radiotherapy planning with total dose of only 50.4 Gy^40^. Therefore, dose escalation with modern radiotherapy techniques and optimal sparing of organs at risk might still be a viable strategy to improve treatment results in esophageal cancer.

A limitation of this study was its retrospective design. The diagnosis of RP is challenging due to differential or concomitant diagnoses such as infections, exacerbation of pre-existing pulmonary conditions or tumor progression that need to be considered. In a trial of Kocak *et al*. scoring of RP done in prospective manner was considered challenging in 28% of lung cancer patients^41^. Even though Hsieh *et al*. report no grade III or IV RP in their retrospective trial, the authors point out in their discussion that eleven of 32 deaths were caused by pneumonia. However, on plan review six of the eleven patients had “pneumonia patches” in the radiation beam pathway. The authors conclude that RP with a complicated course cannot be excluded in these cases^19^. In our trial all cases were evaluated by a multidisciplinary panel of a radiation oncologist, pulmologist and radiologist. Since the treatment of choice of symptomatic RP includes the administration of steroids, we consider it unlikely that cases of symptomatic RP remain undocumented and therefore undetected in our trial. We had excluded patients who had a follow-up of less than three months since the inclusion of these patients could have resulted in an inadequately lower incidence of RP.

In conclusion, both our institutional data and data from 874 patients in our systematic review show a low incidence of symptomatic RP after IMRT for esophageal cancer. Caution is warranted in patients with pre-existing lung conditions, in particular fibrosis. While no relevance was seen for V5, V20 should remain below 23% to keep the likelihood of symptomatic RP below 10%. Future studies using novel techniques for treatment should report DVH parameters together with toxicity data to permit further refinement of existing models for prediction of RP.
